# A Mouse Model for Conditional Secretion of Specific Single-Chain Antibodies Provides Genetic Evidence for Regulation of Cortical Plasticity by a Non-cell Autonomous Homeoprotein Transcription Factor

**DOI:** 10.1371/journal.pgen.1006035

**Published:** 2016-05-12

**Authors:** Clémence Bernard, Clémentine Vincent, Damien Testa, Eva Bertini, Jérôme Ribot, Ariel A. Di Nardo, Michel Volovitch, Alain Prochiantz

**Affiliations:** Centre for Interdisciplinary Research in Biology (CIRB), Collège de France, CNRS UMR 7241/INSERM U1050, PSL Research University, Paris, France; Albert Einstein College of Medicine, UNITED STATES

## Abstract

During postnatal life the cerebral cortex passes through critical periods of plasticity allowing its physiological adaptation to the environment. In the visual cortex, critical period onset and closure are influenced by the non-cell autonomous activity of the Otx2 homeoprotein transcription factor, which regulates the maturation of parvalbumin-expressing inhibitory interneurons (PV cells). In adult mice, the maintenance of a non-plastic adult state requires continuous Otx2 import by PV cells. An important source of extra-cortical Otx2 is the choroid plexus, which secretes Otx2 into the cerebrospinal fluid. Otx2 secretion and internalization requires two small peptidic domains that are part of the DNA-binding domain. Thus, mutating these “transfer” sequences also modifies cell autonomous transcription, precluding this approach to obtain a cell autonomous-only mouse. Here, we develop a mouse model with inducible secretion of an anti-Otx2 single-chain antibody to trap Otx2 in the extracellular milieu. Postnatal secretion of this single-chain antibody by PV cells delays PV maturation and reduces plasticity gene expression. Induced adult expression of this single-chain antibody in cerebrospinal fluid decreases Otx2 internalization by PV cells, strongly induces plasticity gene expression and reopens physiological plasticity. We provide the first mammalian genetic evidence for a signaling mechanism involving intercellular transfer of a homeoprotein transcription factor. Our single-chain antibody mouse model is a valid strategy for extracellular neutralization that could be applied to other homeoproteins and signaling molecules within and beyond the nervous system.

## Author Summary

Classically, cell signaling is based on the secretion of molecules that bind cell surface receptors. Lipophilic agents can do without cell-surface receptors due to their ability to diffuse through the plasma membrane, but this is normally not the case for proteins, which cannot pass the membrane barrier. However, homeoprotein transcription factors represent an exception as they are secreted and internalized by live cells owing to two peptidic domains. An important illustration of this novel signaling mechanism is provided by Otx2, a homeoprotein that travels from the choroid plexus to specific inhibitory neurons in the cerebral cortex, where it regulates physiological plasticity throughout life. Because the two transfer peptides are in the DNA-binding domain of Otx2, it is impossible to mutate them without altering both cell signaling and cell-autonomous functions. We have therefore developed a mouse in which a secreted anti-Otx2 single-chain antibody can be induced to trap extracellular Otx2 while leaving its cell autonomous function untouched. We show that neutralizing extracellular Otx2 modifies the expression of plasticity genes in the visual cortex, thus providing the first genetic demonstration for homeoprotein signaling in a mammal.

## Introduction

During postnatal life, the cerebral cortex passes through critical periods (CPs) of plasticity allowing the neuronal circuitry to shape in response to environmental stimuli. CPs are driven by the maturation of a subset of inhibitory interneurons, the fast-spiking parvalbumin-expressing GABAergic neurons (PV cells), present in layers III and IV of the cerebral cortex [1]. Plasticity terminates with the full maturation of PV cells and the consolidation of the Excitation/Inhibition (E/I) cortical balance [2]. CPs for different sensory, motor or cognitive behaviors are spread out during postnatal development and thus open and close at different times [3]. In the mouse, plasticity for the establishment of binocular vision opens at post-natal day 20 (P20) and closes at P40 [4]. Closing one eye during this period (but not before P20 or after P40) leads to an irreversible loss of visual acuity for the temporarily closed eye, a state known as amblyopia, which affects 3% of the human population.

The transfer of the homeoprotein (HP) transcription factor Otx2 from extra-cortical sources into PV cells of the primary visual cortex (V1) regulates CP timing [5]. Cortical infusion of recombinant Otx2 accelerates both CP onset and closure. Recently, the choroid plexus was identified as one of these sources, as Otx2 knock-down specifically in the choroid plexus reopens a window of plasticity in the adult V1 [6]. Reopening plasticity in the adult was also achieved by blocking Otx2 binding to complex sugars embedded in perineuronal nets (PNNs) that surround PV cells, thus reducing its internalization [7, 8]. Together, these results led to the important concept that Otx2 accumulation by PV cells leads to a first concentration threshold that opens plasticity at P20 and to a second threshold that closes plasticity at P40; the maintenance of a non-plastic adult state requires the continuous internalization of this HP [9, 10].

In this report we aimed at developing genetic evidence for direct non-cell autonomous regulation of plasticity by Otx2 in the mouse. An obvious approach would be the production of a “cell autonomous-only” mouse through the mutation of Otx2 import and/or export sequences. This strategy is precluded by the fact that the latter sequences are part of the DNA-binding domain and cannot be modified without modifying the cell autonomous transcription of Otx2 targets [11, 12]. Based on previous studies on non-mammalian embryonic development [13–16], we turned to a strategy that employs the extracellular expression of neutralizing single-chain variable fragment (scFv) antibodies. We generated conditional knock-in mouse lines (*scFvOtx2*^*tg/o*^ and *scFvPax6*^*tg/o*^) expressing secreted scFv antibodies directed against Otx2 or Pax6 (as a control). Targeted expression of anti-Otx2 scFv in juvenile or adult mice resulted in the delay or reactivation of plasticity genes, respectively. This strategy provides the first genetic evidence for HP transfer in mammals and will allow for precise genetic manipulation of non-cell autonomous homeoprotein activities to study their functions in embryonic and postnatal development.

## Results

**Fig 1:**
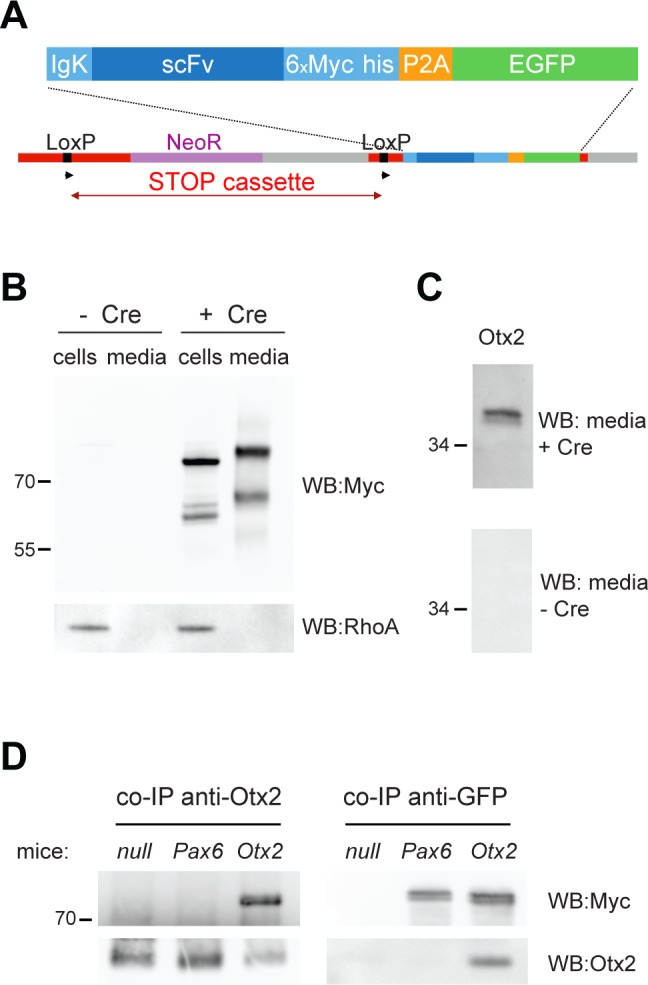
Inducible scFv knock-in mice. **A)** Construct for *scFv* knock-in mouse. The single chain antibody (scFv) is knocked in the *ROSA26* locus of C57Bl6 mice. The scFv plasmid contains an IgK signal peptide for secretion, 6 Myc tags for detection and a skipping peptide (P2A) before EGFP. A STOP cassette is inserted before the scFv construct, flanked by two LoxP sites. **B)** Western blot showing expression (cells) and secretion (media) of anti-Otx2 scFv, upon treatment with Cre-TAT protein of *scFvOtx2*^*tg/o*^ ear fibroblasts. The scFv was revealed with an anti-Myc antibody and RhoA served as an intracellular protein control. **C)** Western blot with recombinant Otx2 protein, revealed with media of *scFvOtx2*^*tg/o*^ fibroblasts treated or not with Cre-TAT. **D)** Co-immunoprecipitation of Otx2 protein and anti-Otx2 scFv from P30 *PV*::*Cre*, *PV*::*Cre;scFvPax6*^*tg/o*^ and *PV*::*Cre;scFvOtx2*^*tg/o*^ cerebellum lysates. Co-immunoprecipitations were performed using Otx2 (left) or GFP (right) antibodies and probed on Western blots using anti-Otx2 or anti-Myc antibodies.

We generated knock-in mouse lines expressing inducible secreted Myc-tagged anti-Otx2 or anti-Pax6 single-chain antibodies (*scFvOtx2*^*tg/o*^ and *scFvPax6*^*tg/o*^) designed to neutralize HPs in the extracellular space without affecting cell autonomous functions. Tagged anti-Otx2 and anti-Pax6 scFv constructs were generated from hybridoma, cloned behind a floxed STOP cassette, and knocked into the *ROSA26a* locus (Fig 1A).

To validate scFv expression, fibroblasts cultured from adult *scFvOtx2*^*tg/o*^ ear biopsies were treated with the cell-permeable Cre bacterial recombinase Cre-TAT [11]. Cells and media were collected 48h later and analyzed by Western blot (Fig 1B). Anti-Otx2 scFv was only present in cells treated with Cre-TAT and in the medium collected from these cells. Intracellular control RhoA confirms cell integrity. The anti-Myc antibody revealed two bands in the cell extract and in the medium. The lower band corresponds to the scFv recombinant protein and the upper band to the scFv-GFP fusion protein, establishing that the P2A skipping peptide was not 100% efficient. The slightly lower mobility of the antibodies retrieved from the extracellular milieu is due to differences in glycosylation [17].

The anti-Pax6 scFv functional activity has been demonstrated earlier [13, 16]. To examine whether secreted anti-Otx2 scFv binds Otx2 specifically, the media of *scFvOtx2*^*tg/o*^ fibroblasts treated or not with Cre-TAT were used as a source of primary antibodies. Otx2 is recognized by the medium of *scFvOtx2*^*tg/o*^ fibroblasts treated with Cre-TAT (Fig 1C). To confirm scFv in vivo activity, we crossed *scFv* mice with *PV*::*Cre* mice, where the Cre recombinase is driven by the PV promoter [18], and extracted adult cerebellum, which expresses both HPs [19] as well as PV. The anti-Otx2 scFv co-immunoprecipitated with Otx2 and, conversely, Otx2 co-immunoprecipitated with the scFv (Fig 1D). Association with Otx2 protein was not found in *PV*::*Cre* or *PV*::*Cre;scFvPax6*^*tg/o*^ cerebellar extracts.

**Fig 2:**
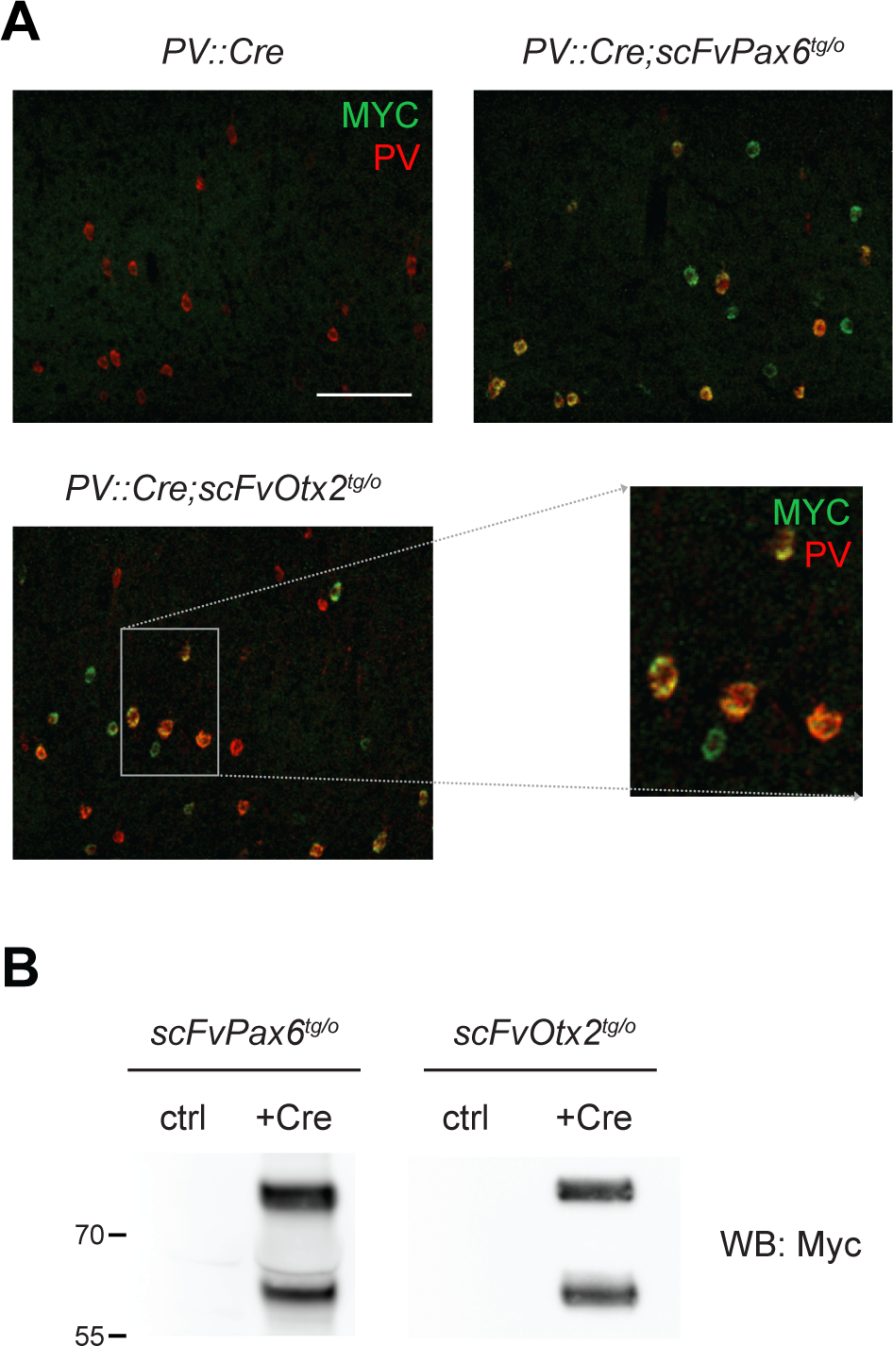
Strategies for inducing expression of scFv in PV cells or in CSF. **A)** Co-staining for Myc and PV in layers II-IV of the binocular zone of the visual cortex (V1b) of P30 *PV*::*Cre*, *PV*::*Cre;scFvPax6*^*tg/o*^ and *PV*::*Cre;scFvOtx2*^*tg/o*^ mice (scale bar: 100μm). The enlarged view shows the immunofluorescence overlap. **B)** Western blot of cerebrospinal fluid (CSF) protein extracts from *scFvOtx2*^*tg/o*^ and *scFvPax6*^*tg/o*^ adult mice injected (+Cre) or not (ctrl) with Cre-TAT protein in lateral ventricles. Anti-Otx2 and anti-Pax6 scFvs (revealed with anti-Myc antibody) are secreted in the CSF upon choroid plexus recombination.

Two models were developed to disrupt non-cell autonomous Otx2 in V1. A first model was based on local scFv secretion by PV cells. A second model consisted in inducing scFv secretion in the cerebrospinal fluid (CSF) from the choroid plexus, which is an endogenous source of extracellular Otx2 [6]. Pax6 is poorly expressed in the postnatal cortex and choroid plexus [20, 21], making *scFvPax6*^*tg/o*^ mice an appropriate negative control. To induce scFv expression by PV cells, *scFv* mice were crossed with *PV*::*Cre* mice, where recombination is driven by the PV promoter starting at around P10 in V1 [1]. At P30, PV cells of *PV*::*Cre;scFvOtx2*^*tg/o*^ and *PV*::*Cre;scFvPax6*^*tg/o*^ mice but not of *PV*::*Cre* mice express scFvs in the V1 binocular zone (Fig 2A and S1 Fig). While most PV cells express the scFv, curiously some cells not stained with the PV antibody also express the scFv. This may result from poor PV expression at P30 or by the capture of secreted scFv by PV-negative cells. To induce scFv expression in the choroid plexus and subsequent secretion into the CSF, adult *scFvPax6*^*tg/o*^ and *scFvOtx2*^*tg/o*^ mice were injected with Cre-TAT in the lateral ventricles [6]. Western blot analysis of the CSF confirmed the induction and secretion of both the scFv and scFv-GFP proteins 5 d post-injection (Fig 2B).

**Fig 3:**
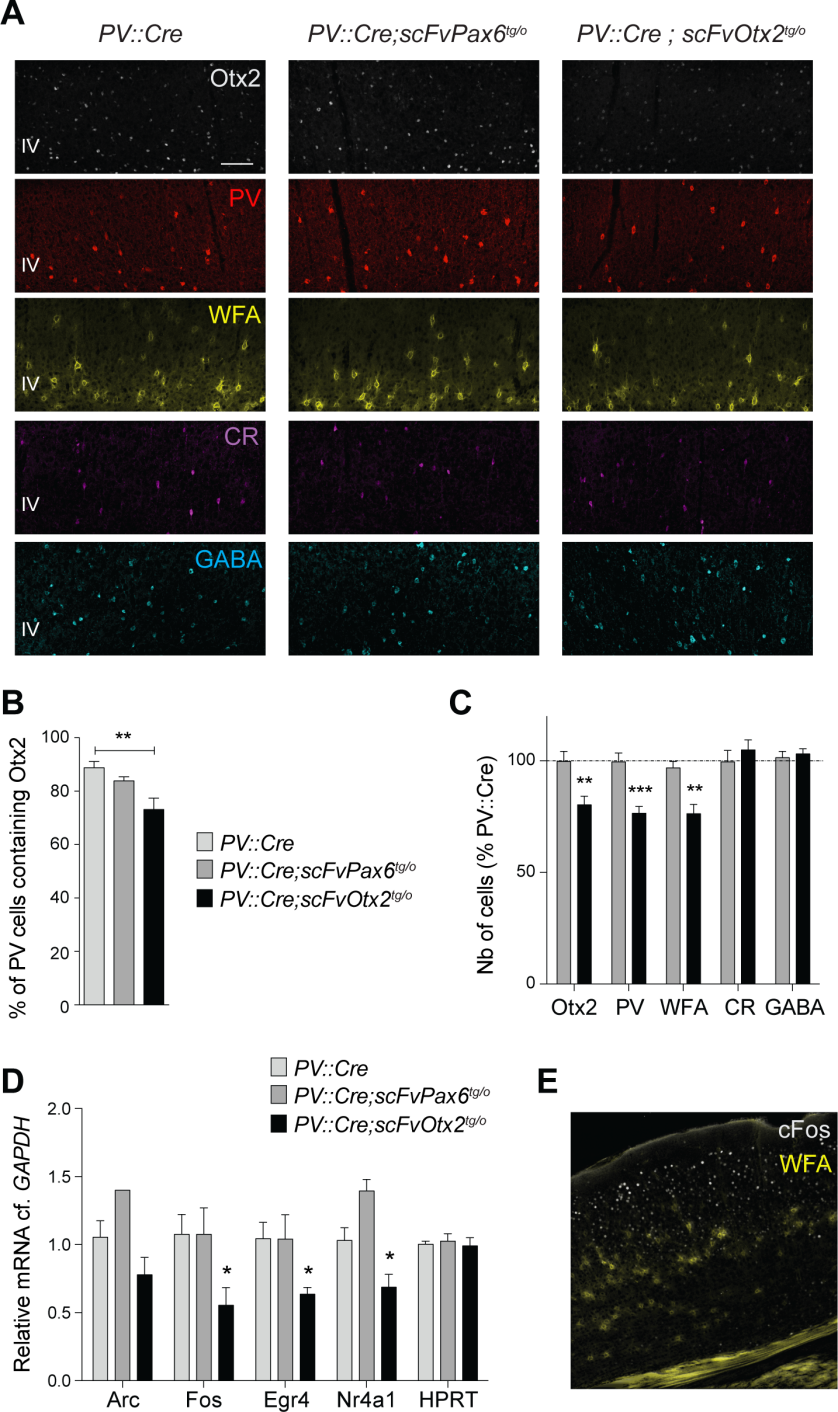
Local expression of anti-Otx2 scFv delays PV cell maturation. **A)** Immunostaining for Otx2, PV, WFA, CR and GABA in V1b layers II-IV of P30 *PV*::*Cre*, *PV*::*Cre;scFvPax6*^*tg/o*^ and *PV*::*Cre;scFvOtx2*^*tg/o*^ mice (scale bar: 100μm). **B)** Percentages of PV-positive cells that contain Otx2 in V1b layers II-IV of P30 *PV*::*Cre*, *PV*::*Cre;scFvPax6*^*tg/o*^ and *PV*::*Cre;scFvOtx2*^*tg/o*^ mice. Absolute cell numbers are provided in S2 Fig. **C)** Quantification of cells stained for Otx2, PV, WFA, CR and GABA in V1b layers II-IV of P30 *PV*::*Cre*, *PV*::*Cre;scFvPax6*^*tg/o*^ and *PV*::*Cre;scFvOtx2*^*tg/o*^ mice (in percentage of *PV*::*Cre* mice, dashed line indicates *PV*::*Cre* levels). Absolute cell numbers are provided in S2 Fig. **D)** Quantification of Arc, c-Fos, Nr4a1, Egr4 and HPRT mRNA contents (relative to GAPDH) in V1b layer IV extracts from P30 *PV*::*Cre*, *PV*::*Cre;scFvPax6*^*tg/o*^ and *PV*::*Cre;scFvOtx2*^*tg/o*^ mice. **E)** Co-staining for c-Fos and WFA in V1b of P30 *PV*::*Cre* mice. (t-tests and one-way ANOVAs; 2–9 mice per group; *p<0.05, **p<0.01, ***p<0.001; error bars indicate SEM)

The accumulation of Otx2 in V1 PV cells controls CP timing through the maturation of PV cells [10]. CP kinetics are paralleled by increasing PV expression and assembly of extracellular matrix perineuronal nets (PNNs) revealed by the WFA lectin [22]. To follow PV cell maturation, P30 V1 sections were stained with antibodies against Otx2 and PV and with fluorescent WFA (Fig 3A). *PV*::*Cre* and *PV*::*Cre;scFvPax6*^*tg/o*^ mice were undistinguishable for all criteria used. In contrast, *PV*::*Cre;scFvOtx2*^*tg/o*^ mice showed a decrease in the number of PV cells containing detectable amounts of Otx2 (Fig 3B). In addition scFv expression reduced by ~20% the number of cells decorated with Otx2 and PV antibodies or with WFA (Fig 3C). Intensity of Otx2 staining was also reduced by 20%. The number of GABAergic neurons and Calretinin (CR) neurons were not modified by scFv induction (Fig 3C and S2 Fig). This possibly reflects limited antibody diffusion and is in agreement with the fact that Otx2 is primarily captured by PV cells and does not influence the maturation of other interneurons [5]. Thus, the anti-Otx2 scFv secretion by PV cells during postnatal development partially blocked Otx2 internalization and delayed PV cell maturation.

Due to the existence of thresholds, small changes in Otx2 concentrations within PV cells can have dramatic effects on V1 plasticity [6, 7]. Plasticity is paralleled by the expression of plasticity genes in V1 layer IV such as *Arc*, *Fos*, *Nr4a1* and *Egr4* [23–28]. Quantitative mRNA analysis of V1 layer IV extracts at P30 demonstrated that the expression of the anti-Otx2 scFv, in addition to delaying PV cell maturation, significantly decreased the up-regulation of *Fos*, *Egr4* and *Nr4a1* transcripts (Fig 3D). *Arc* and *c-Fos* are expressed throughout V1 layers, and *Egr4* and *Nr4a1*, although showing higher levels of expression in V1 layer IV (Fig 3E and S3 Fig), are not necessarily specifically expressed in PV cells. Thus, changes in layer IV gene expression reflect the global effect on plasticity caused by Otx2 reduction.

**Fig 4:**
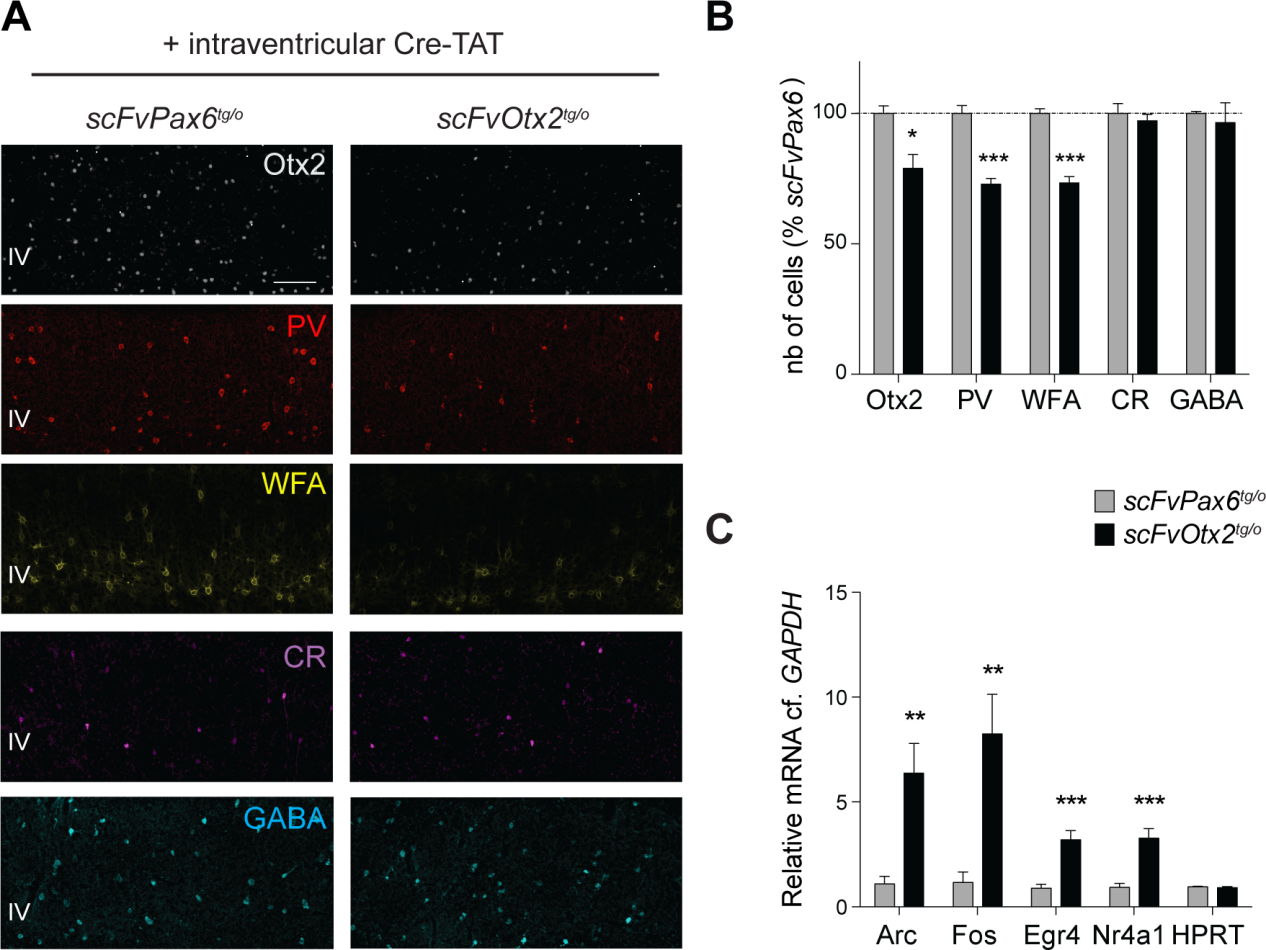
Anti-Otx2 scFv in adult CSF activates plasticity genes. **A)** Immunostaining for Otx2, PV, WFA, CR and GABA in V1b layers II-IV of adult *scFvPax6*^*tg/o*^ and *scFvOtx2*^*tg/o*^ mice, 15 days after intracerebroventricular (icv) injection of Cre-TAT protein (scale bar: 100μm). **B)** Quantification of number of Otx2-, PV-, WFA, CR- and GABA-positive cells in V1b layers II-IV of adult *scFvOtx2*^*tg/o*^ and *scFvPax6*^*tg/o*^ mice, 15 days after icv injection of Cre-TAT (in percentage of *scFvPax6*^*tg/o*^ mice, dashed line indicates *scFvPax6*^*tg/o*^ levels). Absolute numbers are provided in S2 Fig. **C)** Quantification of *Arc*, *Fos*, *Nr4a1*, *Egr4* and *HPRT* mRNA content (relative to *GAPDH*) in V1 layer IV extracts from adult *scFvOtx2*^*tg/o*^ and *scFvPax6*^*tg/o*^ mice, 15 d after icv injection of Cre-TAT. (t-tests; 3–12 mice per group; *p<0.05, **p<0.01, ***p<0.001; error bars indicate SEM)

Adult choroid plexus expression of scFv was induced in P90-P120 *scFvOtx2*^*tg/o*^ and *scFvPax6*^*tg/o*^ mice. After 15 d, the number of cells expressing Otx2, PV or stained with WFA (PNN assembly) was analyzed. In binocular V1 of injected *scFvOtx2*^*tg/o*^ compared to injected *scFvPax6*^*tg/o*^, the number of cells stained with Otx2 as well as the number of cells expressing the two markers of PV cell maturation were reduced by 20 to 30%, with no change in the percentage of CR neurons or of the entire population of GABAergic neurons (Fig 4A and 4B and S2 Fig). In parallel, plasticity transcript expression was dramatically enhanced in V1 layer IV (Fig 4C), suggesting induced brain plasticity similar to the effect obtained by recombination of *Otx2* in the adult choroid plexus [6].

**Fig 5:**
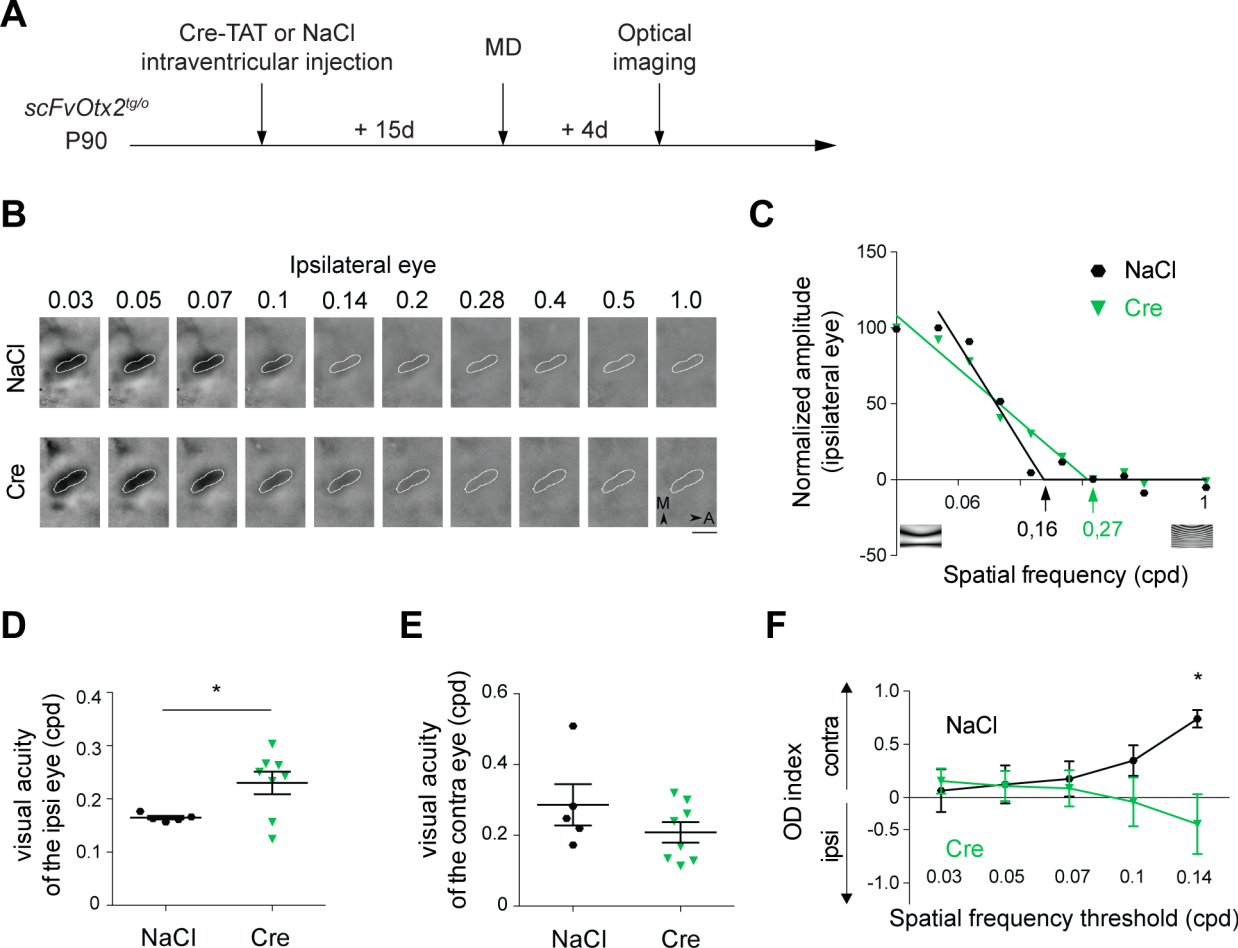
Anti-Otx2 scFv in adult CSF restores ocular dominance plasticity. **A)** *scFvOtx2*^*tg/o*^ adult (P90) mice were injected with NaCl or Cre-TAT and, after 15 d, the left eye was sutured for 4 d. Intrinsic optical imaging was then performed in the right visual cortex to measure visual acuity for each eye. **B)** Intrinsic signals recorded for the ipsilateral eye stimulation in *scFvOtx2*^*tg/o*^ adult mice injected with NaCl (top) or Cre-TAT (bottom). Ten spatial frequencies (SFs) ranging from 0.03 to 1 cycle/degree were tested. The binocular zone is delineated in white (A, Anterior; M, Medial; Scale bar, 1 mm). **C)** Estimation of the ipsilateral eye visual acuity for the two mice shown in (B). Each point corresponds to the average magnitude in the binocular zone. For each animal, data were normalized by the maximal amplitude among SFs and visual acuity was defined as the intersection of a fitted line segment with the X-axis. Representative stimuli are shown below. **D)** Visual acuity of the ipsilateral eye in adult *scFvOtx2*^*tg/o*^ injected with NaCl or Cre-TAT. **E)** Same as (D) for the contralateral eye. **F)** Ocular dominance index at different SF thresholds in adult *scFvOtx2*^*tg/o*^ mice injected with NaCl or Cre-TAT. Positive values indicate a preference for the contralateral eye. (t-tests; *p<0.05; error bars indicate SEM)

To verify if the reactivation of plasticity genes was reflected at a physiological level, we used optical imaging of intrinsic signals to measure visual acuity [29, 30]. Two weeks after intra-ventricular injection of Cre-TAT or NaCl, adult (P105) *scFvOtx2*^*tg/o*^ mice underwent 4 d of monocular deprivation (MD) prior to optical imaging in the opposite hemisphere (Fig 5A). The responses to different spatial frequencies were measured and quantified, as illustrated in Fig 5B and 5C. The expression of anti-Otx2 scFv enhances visual acuity of the ipsilateral eye (Fig 5D) but does not modify that of contralateral eye (Fig 5E), leading to a shift in ocular dominance for the highest spatial frequencies (above 0.14 cpd, Fig 5F).

## Discussion

The possibility of the anti-Otx2 scFv neutralizing cell autonomous Otx2 cannot be ignored even though the amount of antibody that might escape the secretion pathway is low and the disulfide bond necessary for antibody activity is reduced in the intracellular milieu [31, 32]. This risk can be excluded when PV cells express the antibody, given that the Otx2 locus is not active in these cells. In contrast, the issue can be raised for the choroid plexus, which strongly expresses Otx2. However, we have verified that potential Otx2 target genes in the choroid plexus are not quantitatively modified in the recombined *scFvOtx2*^*tg/o*^ plexus. For example an identified target gene is C1qL3 which, compared to GAPDH, is up-regulated from 1.01 ± 0.11 (n = 3) to 5.21 ± 0.62 (n = 3) in the plexus of *Otx2*^*+/-*^ mice and unchanged 1.40 ± 0.07 (n = 3) in the scFv mouse choroid plexus.

The consequences of expressing the anti-Otx2 scFv under the control of the PV promoter are only a modest reduction of Otx2 content, PV expression and PNN assembly at P30. One likely reason is that PV expression starts at around P10 [1] and that it might take some time before the concentration of extracellular antibody is high enough to completely block Otx2 internalization. Another possibility is that the high affinity (in the nM range) of Otx2 for PNN complex sugars [7] partially protects Otx2 from being neutralized by the antibody. It is nevertheless clear that the delayed time course in PV cell maturation is enough to delay the expression of plasticity genes.

A similarly modest reduction in cortical Otx2 content occurred after anti-Otx2 scFv expression was induced in the adult choroid plexus. Nonetheless, the level of reduction is comparable to what was previously obtained when Otx2 expression was knocked down in the adult choroid plexus, which resulted in the reopening of V1 binocular plasticity [6]. This suggests that the antibody complexes most of the extracellular transcription factor in the CSF. The remaining Otx2 still present in PV cells after 2 weeks either reflects the stability of protein present before scFv expression or the existence of sources other than the choroid plexus, in particular the eye [5, 33] and the pineal gland, another established site of Otx2 expression [34]. Regardless, this modest reduction is enough to induce a dramatic re-expression of plasticity genes in the adult and an enhancement of visual cortex plasticity. The plasticity observed after 4 d of MD in P105 mice corresponds to an increase in ipsilateral visual acuity with no change to the deprived contralateral eye. Indeed, Sato & Stryker [30] previously showed that adult plasticity mainly impacts the response of the non-deprived eye. Furthermore, they showed that 7 d of MD were necessary to induce moderate plasticity in the cortex of adult (P90) mice, while only 4 d were required to induce strong plasticity in juvenile (P30) mice. Thus, Otx2 capture by the scFv antibody in adult (P105) mice accelerates ocular dominance plasticity.

Intercellular transfer has been observed in vivo and/or in vitro for many HPs and the conservation of the transfer sequences in the DNA-binding domain suggests that this is a general property of this transcription factor family. Our single-chain antibody strategy brings clear genetic evidence for Otx2 signaling and will be generally useful to verify HP transport in vivo and to analyze the roles of this novel mode of signal transduction. This same strategy could indeed be applied to the neutralization of any signaling entity within and beyond the nervous system.

This report demonstrates that the extracellular neutralization of Otx2 by the inducible expression of an anti-Otx2 single-chain antibody leads to a reduction in Otx2 capture by PV cells and either delays their maturation or “rejuvenates” them depending on when the antibody is expressed. In both cases, several genes associated with a plastic state are regulated in the expected direction. This provides the genetic evidence for a novel signaling mechanism operating through homeoprotein transfer in the mouse.

## Materials and Methods

### Ethics statement

All animal procedures, including housing, were carried out in accordance with the recommendations of the European Economic Community (86/609/EEC), the French National Committee (87/848) and French bylaws (AGRG1240332A / AGRG1238724A / AGRG1238767A / AGRG1238729A / AGRG1238753A). For surgical procedures, animals were anesthetized with Xylazine (Rompun 2%, 5mg/kg) and Ketamine (Imalgene 500, 80mg/kg). For biochemical analysis, mice were sacrificed by cervical elongation. This research (project no. 00704.02) was approved by Ethics committee n° 59 of the French Ministry for Research and Higher Education.

### scFv construction

Hybridoma expressing monoclonal antibodies (CD4 and CD7) against Otx2 were generated, and CD7 hybridoma mRNAs were used to construct scFv minigenes as described in [35]. CD7-scFv coding sequence was subcloned as described in [16], with an N-terminal signal sequence and C-terminal 6xMyc and His tags followed by a skipping peptide (P2A)-eGFP cassette for expression of either a fusion protein (scFv-myc-his-P2A-GFP) or two separate proteins, depending on P2A peptide efficiency. Anti-Pax6 scFv [13] was transferred into the same final vector.

### Animals

*scFvOtx2* and *scFvPax6* knock-in mouse lines were generated by the Institut Clinique de la Souris (Strasbourg, France) by transgenesis with targeted insertion in the *Rosa26* allele. PV-Cre mice were obtained from The Jackson Laboratory (stock 8069).

### Cell culture of ear fibroblasts

Fibroblasts were generated from ear biopsies from adult *scFvPax6*^*tg/o*^ and *scFvOtx2*^*tg/o*^ mice and treated with 10 μM cell-permeable Cre (Cre-TAT, production described in [6]) in DMEM [11]. Cells and culture media were collected 48 h later and processed for Western blot analysis. Conditioned media were also used as primary antibodies on Western blots.

### Cre-TAT protein injection

Stereotaxic injection (bregma: x = -0.58 mm, y = ±1.28 mm, z = 2 mm) of Cre-TAT in 15% DMSO into the lateral ventricles of anesthetized mice was performed with a Hamilton syringe at a rate of 0.2 μl/min. Five or fifteen days after injection, mice were processed for cerebrospinal fluid sampling (described in [6]), cortical layer dissection or immunohistochemistry.

### Layer IV dissection

The dissection of tissue enriched in cortical layer IV was performed in ice cold PBS with micro scalpels. Visual cortex areas were first excised from both brain hemispheres and then cut coronal to produce two thin rectangular strips per hemisphere. These strips were laid on their sides to expose the cortical layers. A lengthwise cut in two equal parts gave the supragranular layer, which was then cut lengthwise in two equal parts to provide enriched layer IV.

### Immunoprecipitation

Whole cerebellum was dissected and suspended in immunoprecipitation lysis buffer (20 mM Tris pH8, 120 mM NaCl, 1% NP-40, 1 mM MgCl2, 5% glycerol, Benzonase nuclease and protease inhibitors). Samples were centrifuged (10 min, 20 000 g) at 4°C and the supernatant was incubated overnight at 4°C with rotation with anti-Otx2-coupled Dynabeads (using anti-Otx2 rabbit polyclonal, Abcam ab21990) or with anti-GFP-coupled magnetic beads (Chromotek). The beads were washed with lysis buffer and with 1 M urea before Western blot analysis.

### Western blot

Protein extracts were separated on NuPAGE 4–12% Bis-Tris pre-cast gels (Invitrogen) for 1 h at 200 V and transferred onto a methanol-activated PVDF membrane at 400 mA for 1 h. The following primary antibodies were used: anti-Myc (rabbit polyclonal, 1/4000, Sigma-Aldrich C3956), anti-Otx2 (rabbit monoclonal, 1/2000, Abcam ab92326), anti-RhoA (mouse monoclonal, 1/200, Santa Cruz sc418). Membranes were imaged with a LAS-4000 gel imager (Fujifilm) and quantified by densitometry with ImageJ.

### Immunohistochemistry

Mice were perfused transcardially with PBS followed by 4% paraformaldehyde prepared in PBS. Brains were post-fixed 1 h at 4°C and immunohistochemistry was performed on cryosections (20 μm) encompassing the entire visual cortex. Primary antibodies included anti-Myc (rabbit, 1/400, Sigma-Aldrich C3956), anti-Otx2 (mouse monoclonal, in house), anti-PV (rabbit, 1/500, Swant PV25), anti-CR (mouse monoclonal, 1/500, Swant), anti-GABA (rabbit, 1/300, Sigma), and anti-cFos (rabbit monoclonal, 1/300, Cell Signaling). Secondary antibodies were Alexa Fluor-conjugated (Molecular Probes). Biotinylated WFA (1/100, Sigma-Aldrich L1516) with Alexa Fluor-conjugated streptavidin was used to reveal perineuronal nets. Sections were mounted in Fluoromount (Southern Biotech). Images were acquired with a Leica SP5 confocal microscope and analyzed with ImageJ.

### In situ hybridization

In situ hybridization on cryosections (20 μm) were performed as previously described [36]. Briefly, hybridization was carried out overnight at 70°C with DIG-labeled probes. Sections were washed and incubated with alkaline phosphatase-conjugated anti-DIG (1/2000, Roche) overnight at 4°C. Sections were then washed and the color reaction was carried out at room temperature. Reaction was stopped by PBS washes and sections were mounted with Fluoromount (Southern Biotech). Mosaïc images (10x magnification) were acquired with a Nikon 90i microscope.

### Quantitative PCR

Layer IV of visual cortex was manually dissected and RNA was extracted using the RNeasy Lipid Tissue Mini (Qiagen) according to manufacturer’s instructions. cDNA was synthesized from 500 ng of total RNA with the QuantiTect Reverse Transcription kit (Qiagen). For real-time PCR, triplicates were analyzed with a LightCycler 480 II (Roche). Gene-to-GAPDH ratios were determined using the 2^-∆∆Ct^ method.

### Monocular deprivation and surgery for optical imaging

For monocular deprivation, mice were anesthetized prior to suturing of the left eye as described in [37]. Animals were checked daily to ensure sutures remained intact. The eye was opened immediately before recording.

For optical imaging, mice were anesthetized with urethane (1.2 g/kg, i.p.) and a sedative (chlorprothixene, 8 mg/kg i.m.). Atropine (0.1 mg/kg) and dexamethasone (2 mg/kg) were injected subcutaneously and body temperature was maintained at 37°C. Mice were placed in a stereotaxic instrument and craniotomy was made over the primary visual cortex in the hemisphere contralateral to the deprived eye. The exposed area was covered by 2.5% agarose and a glass coverslip.

### Visual stimulation

Visual stimuli were randomly displayed on a 21” LCD monitor located 20 cm in front of the animal. Coordinates of the monitor were reprogrammed in order to maintain spatial and temporal frequencies constant across the visual field’s eccentricities (See examples in Fig 5C, [38]). Each stimulus consisted of oriented sine-wave gratings drifting in one direction perpendicular to the orientation at a temporal frequency of 1.5 Hz. Ten spatial frequencies (SFs) were tested, ranging from 0.0303 to 1 cycle per degree (cpd) in a logarithmic scale. Monocular stimuli were displayed for 5s. A grey screen was presented between trials for 10s. Each SF was displayed 16 times with a different orientation/direction.

### Intrinsic signal optical imaging

Optical images of intrinsic signals were recorded with a Dalsa 1M60 CCD camera controlled with an Imager 3001 system (Optical Imaging Ltd). Images were acquired after a 2x2 pixels spatial binning with a resolution of ∼ 10.5 μm/pixel using a 135x50 mm tandem lens (Nikon) configuration. The focal plane was set 400 μm below the cortical surface, and intrinsic signals were acquired with a 700 nm illumination wavelength. The size of the recorded region was ∼ 2.5 x 3.6 mm. Five frames of 1s duration each were stored right after the stimulus onset.

For data analysis, the last three recorded frames, which correspond to the strongest intrinsic signal, were averaged and divided by the first recorded frame. The generalized indicator function method was applied to identify the stimulus-related activity patterns [38]. An additional low-pass filter with a Gaussian kernel of 3 pixels SD was applied to smooth the data. The ipsilateral eye was stimulated, the area of interest was delineated over the primary visual cortex and the SF tuning curve in the binocular zone was calculated by averaging the response for each SF stimulus. This curve was least-square fitted to extract visual acuity. For each eye the response integral (trapezoidal approximation) elicited by SFs was calculated. Ocular dominance values ranged between -1 and 1 (ipsilateral and contralateral eye response, respectively) and calculated for each SF threshold with the contrast formula from the response integral of each eye.

### Statistical analysis

Statistical analysis was performed with Prism software (GraphPad). Pairwise comparison was done using Student t-test.

## Supporting Information

S1 Fig Anti-Otx2 scFv expression in V1. Co-staining for Myc and PV in visual cortex of a P30 *PV*::*Cre;scFvOtx2*^*tg/o*^ mouse (scale bar: 200µm). The third panel shows the immunofluorescence overlap.
(TIF)

S2 Fig Total number of cells in V1b for both scFv strategies. **A)** Quantification of number of Otx2-, PV-, WFA-, CR- and GABA-positive cells in V1b layers II-IV of P30 *PV*::*Cre;scFvPax6*^*tg/o*^ *and PV*::*Cre;scFvOtx2*^*tg/o*^ mice (one-way ANOVAs; 2–9 mice per group; **p<0.01, ***p<0.001; error bars indicate SEM). **B)** Quantification of number of Otx2-, PV-, WFA, CR- and GABA-positive cells in V1b layers II-IV of adult *scFvOtx2*^*tg/o*^ and *scFvPax6*^*tg/o*^ mice, 15 days after intracerebroventricular injection of Cre-TAT (t-tests; 3–12 mice per group; *p<0.05, ***p<0.001; error bars indicate SEM).
(TIF)

S3 Fig Localization of Arc, Erg4 and Nr4a1 expression in V1b by in situ hybridization. The left panels show the staining for sense probes and the middle panels the staining for antisense probes. The right panel shows WFA immunostaining in V1b to visualize layer IV.
(TIF)
