# Supplementary figures and images for: A Mouse Model for Conditional Secretion of Specific Single-Chain Antibodies Provides Genetic Evidence for Regulation of Cortical Plasticity by a Non-cell Autonomous Homeoprotein Transcription Factor
Source: PLoS Genet. 2016 May 12;12(5):e1006035. doi: 10.1371/journal.pgen.1006035 (PMC4865174; doi:10.1371/journal.pgen.1006035)

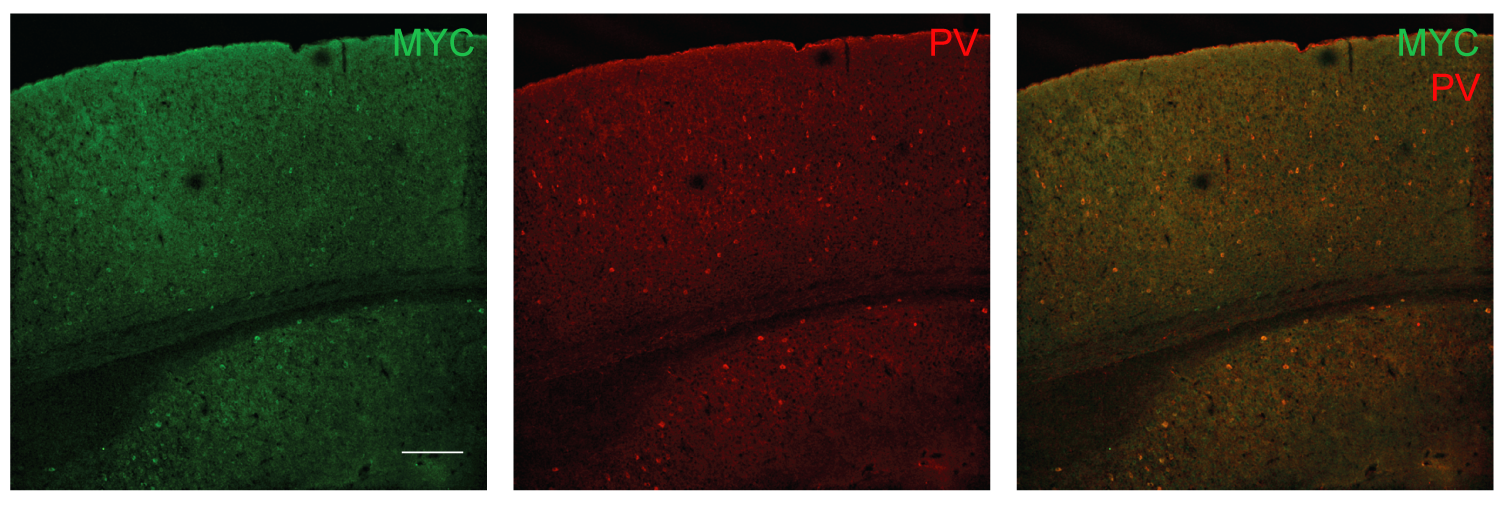

Supplement: S1 Fig — Co-staining for Myc and PV in visual cortex of a P30 PV::Cre;scFvOtx2tg/o mouse (scale bar: 200µm). The third panel shows the immunofluorescence overlap. (TIF) [file pgen.1006035.s001.tif]

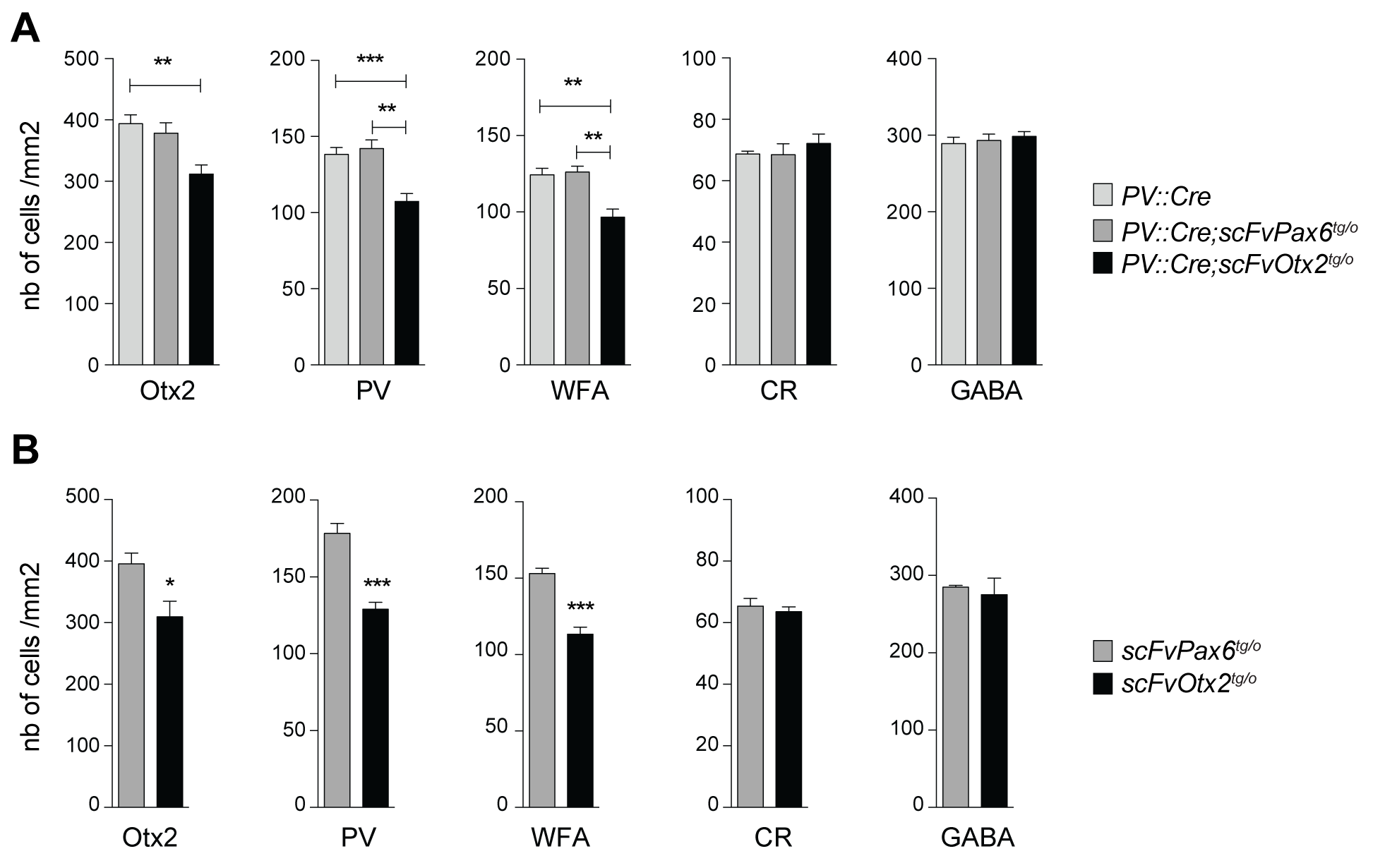

Supplement: S2 Fig — A) Quantification of number of Otx2-, PV-, WFA-, CR- and GABA-positive cells in V1b layers II-IV of P30 PV::Cre;scFvPax6tg/o and PV::Cre;scFvOtx2tg/o mice (one-way ANOVAs; 2–9 mice per group; **p<0.01, ***p<0.001; error bars indicate SEM). B) Quantification of number of Otx2-, PV-, WFA, CR- and GABA-positive cells in V1b layers II-IV of adult scFvOtx2tg/o and scFvPax6tg/o mice, 15 days after intracerebroventricular injection of Cre-TAT (t-tests; 3–12 mice per group; *p<0.05, ***p<0.001; error bars indicate SEM). (TIF) [file pgen.1006035.s002.tif]

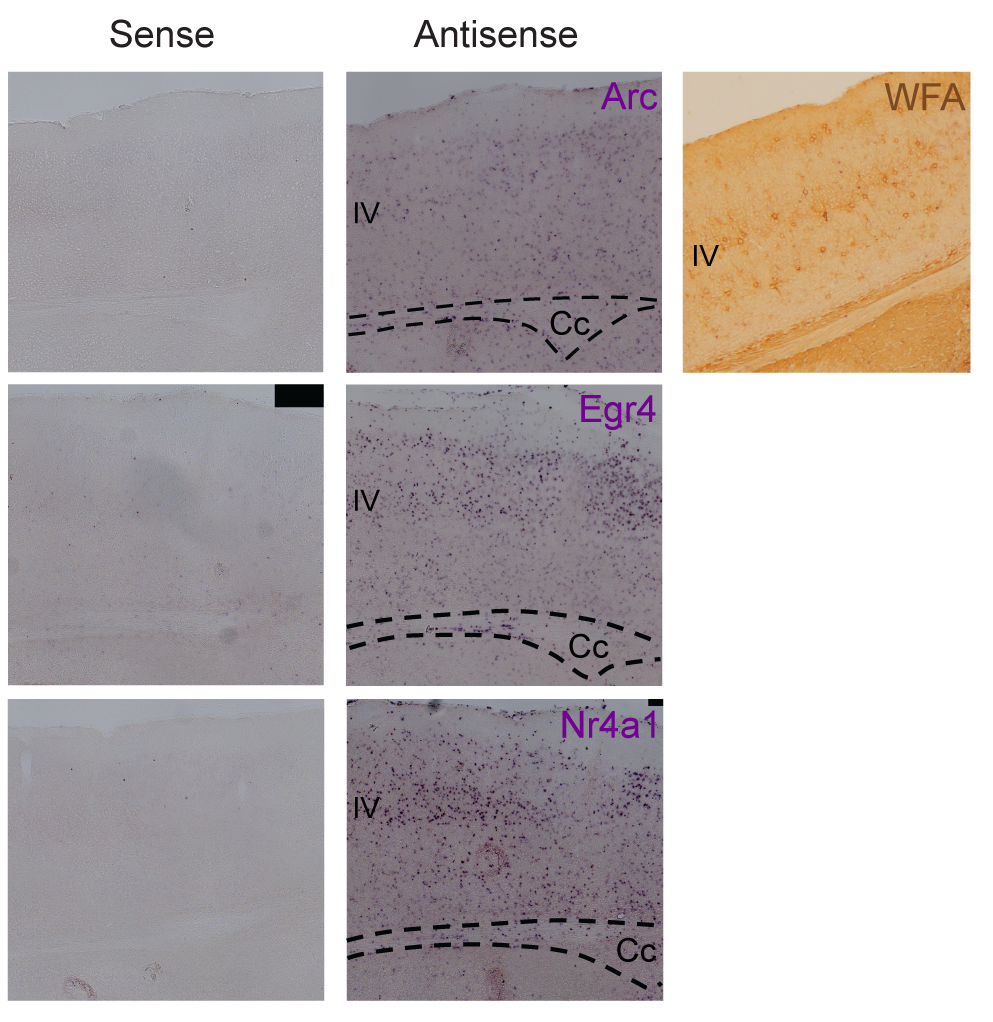

Supplement: S3 Fig — The left panels show the staining for sense probes and the middle panels the staining for antisense probes. The right panel shows WFA immunostaining in V1b to visualize layer IV. (TIF) [file pgen.1006035.s003.tif]
